# Synthesis of a Novel Thiazolidinedione and Evaluation of Its Modulatory Effect on IFN-****γ****, IL-6, IL-17A, and IL-22 Production in PBMCs from Rheumatoid Arthritis Patients

**DOI:** 10.1155/2013/926060

**Published:** 2013-09-01

**Authors:** Laurindo Ferreira da Rocha Junior, Moacyr Jesus Barreto de Melo Rêgo, Mariana Brayner Cavalcanti, Michelly Cristiny Pereira, Marina Galdino da Rocha Pitta, Priscilla Stela Santana de Oliveira, Sayonara Maria Calado Gonçalves, Angela Luzia Branco Pinto Duarte, Maria do Carmo Alves de Lima, Ivan da Rocha Pitta, Maira Galdino da Rocha Pitta

**Affiliations:** ^1^Serviço de Reumatologia do Hospital das Clínicas (HC), Universidade Federal de Pernambuco (UFPE), Rua Tereza Amélia, s/n, Cidade Universitária, 50670-901 Recife, PE, Brazil; ^2^Laboratório de Imunomodulação e Novas Abordagens Terapêuticas (LINAT), Núcleo de Pesquisa em Inovação Terapêutica Suely Galdino (NUPIT-SG), Universidade Federal de Pernanbuco, (UFPE), Rua Tereza Amélia, s/n, Cidade Universitária, 50670-901 Recife, PE, Brazil; ^3^Laboratório de Planejamento e Síntese de Fármacos (LPSF), NUPIT-SG, Universidade Federal de Perambuco, (UFPE), Rua Tereza Amélia, s/n, Cidade Universitária, 50670-901 Recife, PE, Brazil

## Abstract

Rheumatoid arthritis (RA) is an autoimmune disease frequently characterized by chronic synovitis of multiple joints. The pathogenesis of RA is complex and involves many proinflammatory cytokines as Th17 related ones. PPAR**γ** is a nuclear receptor activator that represses proinflammatory gene expression. Thus, this work aimed to synthetize a new thiazolidinedione (TZD) analogue based on a well-known anti-inflammatory and PPAR**γ** agonist activity of this ring and evaluate its anti-inflammatory activity. After chemical structure confirmation, the compound named 5-(5-bromo-2-methoxy-benzylidene)-3-(2-nitro-benzyl)-thiazolidine-2,4-dione TM17 was submitted to cytokine releasing inhibition and PPAR**γ** genetic modulation assays. The new compound showed no toxicity on human and murine cells, decreasing IL-6 secretion by murine splenocytes and reducing IL-17A, IL-22, and IFN-**γ** expression in peripheral blood mononuclear cells from patients with RA. TM17 was more efficient in modulating the mRNA expression of PPAR**γ** than its well-used TZD agonist rosiglitazone. Surprisingly, TM17 was efficient on IL-17A and IFN-**γ** reduction, like the positive control methylprednisolone, and presented a better effect on IL-22 levels. In conclusion, PBMCs obtained from RA patients under TM17 treatment present a significant reduction in IL-17A, IL-22, and IFN-**γ** levels, but not IL-6 when compared with nontreated cells, as well as increase PPAR**γ** mRNA expression in absence of stimulus addressing it as a promising molecule in RA treatment.

## 1. Introduction

Rheumatoid arthritis (RA) is a chronic autoimmune disease that is associated with systemic complications and early death [1–3]. RA is characterized by synovial inflammation, autoantibody production, cartilage and bone destruction, and extraarticular features. The disease leads to deformity and the inflammatory burden is associated with cardiovascular, pulmonary, psychological, and skeletal disorders [1, 2]. The pathogenesis of RA is complex and involves T cells, B cells, and the interaction of many proinflammatory cytokines mainly of Th1 and Th17 pathways [3–6].

Previous studies have demonstrated the anti-inflammatory properties of peroxisome proliferator-activated receptor-gamma (PPAR*γ*) agonists, in experimental models of arthritis and in various inflammatory cells [7, 8]. The PPAR*γ* is a nuclear receptor that plays key roles in the regulation of metabolic homeostasis and inflammation [9]. Its activation in immune cells predominantly results in repression of proinflammatory gene expression like TNF, IL-1B, and IL-6 [10–15].

Many ligands that activate and modulate PPAR functions have been identified [16]. The thiazolidinediones (TZDs), a class of antidiabetic drugs, function as high-affinity PPAR*γ* ligands. The thiazolidines-2,4-diones (TZDs) have been extensive researched due to their deep involvement in regulation of different physiological processes like cell proliferation, angiogenesis, inflammation, and glucose metabolism [17] as wells as a strong association with the inhibition of T-cell activation and inflammatory disease [18]. Thus, these classes of drugs are of growing importance as a therapeutical approach in inflammatory and autoimmune diseases such as RA. This work aimed to evaluate the immunomodulatory activity of a new TZD analogue called TM17 in RA patients cells.

## 2. Materials and Methods 

### 2.1. Anti-Inflammatory Assay

#### 2.1.1. Animals

Experimental assays utilized BALB/c mice (male, 45 days old). The animals (*n* = 6) were raised and maintained at the animal facilities of the Laboratory of Imunopatologia Keizo Asami (LIKA) (Universidade Federal de Pernambuco, Recife, Brazil). All mice were killed and treated in accordance with the guidelines of the Ethical Committee for the Use of Experimental Animals of the Universidade Federal de Pernambuco. For splenocytes obtention the spleen was extracted aseptically and placed in a Petri dish containing RPMI-1640 (Gibco). In a vertical flow, each spleen was transferred to another Petri dish where they were submerged. The cell suspension obtained from each spleen was filtered in a cell sytrainer 40 *μ*m nylon (BD FalconTM) and then transferred to Falcon tubes. The spleen concentrates were then centrifuged twice for 10 minutes. Subsequently the cells were lysed with RBC lysis buffer 1X (eBiosciences) and ressuspended at in RPMI-1640 (Sigma) medium supplemented with 10% fetal bovine serum, 10 mM HEPES (4-(2-hydroxyethyl)-1-piperazineethanesulfonic acid) (Gibco), and 200 U/mL penicillin/streptomycin (Gibco). The cell viability was determined by trypan blue 0.4% (Sigma-Aldrich, USA) exclusion at 1 : 4 dilution (1 part of cells : 4 parts of dye). The samples were only used when viability was >98%.

#### 2.1.2. AR Patients and Health Voluntaries

Patients with RA (*n* = 9) were recruited from Rheumatology Division at Hospital das Clinicas-Universidade Federal de Pernambuco. Demographic, clinical, current medication, and laboratorial data were collected from all patients by questionnaire and from hospital records (Table 1). Patients were included after fulfilling at least four or more of the American College of Rheumatology (ACR) 1987 classification criteria for RA [19]. After exclusion of any rheumatic disease healthy volunteers were recruited as a control group (*n* = 9). Peripheral blood samples were obtained from patients and healthy volunteers. Informed written consent was obtained from all patients and controls in agreement with the norms of the Health Science Center Ethical Committee. The peripheral blood mononuclear cells (PBMCs) were isolated from blood of health donors and patients with RA by centrifugation on Ficoll PaqueTM Plus (density 1.077 g/mL -GE Healthcare Bio-Sciences). Then, the PBMCs were ressuspended in RPMI 1640 medium (Gibco) supplemented with L-glutamine, 10% fetal serum bolvino (Gibco), 10 mM HEPES (4-(2-hydroxyethyl)-1-piperazineethanesulfonic acid) (Gibco) and 200 U/mL penicillin/streptomycin (Gibco). The cell viability was determined by trypan blue 0.4% (Sigma-Aldrich, USA) exclusion at 1 : 4 dilution (1 part of cells : 4 parts of dye). The samples were only used when viability was >98%. 

#### 2.1.3. MTT Assay

The splenocytes from BALB/c mice and PBMCs from health donors were incubated in the presence of two different concentrations (100 and 250 *μ*M for splenocytes and 10 and 100 *μ*M for PBMCs) of the compounds for 48 hours. Then cytotoxicity was quantified by the ability of living cells to reduce MTT to a purple compound. The compounds were tested in three independent assays, and at the end of incubation wells were centrifuged, and the medium was replaced by medium without compound (150 *μ*L) containing MTT (0.5 mg/mL). Three hours later the MTT formazan was diluted with 100 mL of 20% SDS, and its absorbance was measured (570 nm) by the apparatus (BioTek EL808). The cytotoxic activity was quantitated as the percentage of control absorbance. In this sense, the absorbance of the TM17 treated group was obtained in relation to vehicle treated control group. In all analyzed experiments, the vehicle (DMSO 0.1%) treated group presented viability >97% compared to cells control without vehicle.

#### 2.1.4. PBMCs and Splenocytes Cultures

PBMCs (1 × 10^6^ cell/mL) and splenocytes (2 × 10^6^ cell/mL) were cultured in RPMI-1640 (Gibco) supplemented with 10% fetal bovine serum (Gibco), HEPES 10 mM (Gibco), and penicillin (10.000 U/mL)/(streptomycin 10.000 *μ*g/mL) (Gibco). Cells were stimulated with PMA (Sigma) + ionomycin (Sigma) for PBMCs or with Conocavalia-A (ConA) for splenocyte, in the presence or absence of TM-17 at concentrations of 1, 10, and 100 *μ*M. Methyprednisolone 100 *μ*M (Pfizer) was used as a positive control. Cells were incubated for 48 hours at 37°C in humidified 5% CO_2_ incubator.

#### 2.1.5. Cytokine Titration

Cytokines of cultures supernatants were determined by enzyme-linked immunosorbent assay (ELISA) kits according to the manufacturer's instructions. In culture supernatants from PBMCs, IL-6 (BD Biosciences), IFN-*γ* (BD Bioscience), IL-17A (R&D Systems) and IL-22 (eBiosciences) were determined. The lower limits of detection for the ELISA analyses were as follows: 15.625 pg/mL for human IL-17, 9.375 pg/mL for human IL-6 and IFN-*γ*, and 31.25 pg/mL for human IL-22.

#### 2.1.6. Quantitative RT-PCR

Total RNA from cells was extracted using trizol (Life Technologies) according to manufacturer's instructions. Next, cDNA synthesis was obtained from 3-4 *μ*g of total RNA using the High Capacity Archive kit (Applied Biosystems). PPAR*γ* mRNA levels were measured by real time PCR using 18S ribosomal gene as the internal standard. Standard TaqMan probes were Hs01115513_m1 for PPAR*γ* and Hs03928990_g1 for 18S amplification. Real-time PCR reactions were performed on ABIPrism 7900HT sequence detection PCR machine (Applied Biosystem) according to the manufacturer's protocol. The relative gene expression was calculated by 2^−ΔΔCT^.

#### 2.1.7. Statistical Analysis

All experiments were performed at least three independent times before statistical analysis, and the results in this were analyzed by univariate comparisons using nonparametric tests (Wilcoxon matched pairs test) with *P* < 0.05 being considered as a significant difference. All quantitative data were plotted with GraphPad Prism 3.02 software and in all graphs; bars represent mean value ± standard deviation.

## 3. Results and Discussion

### 3.1. Chemical Synthesis

**Figure 1:**
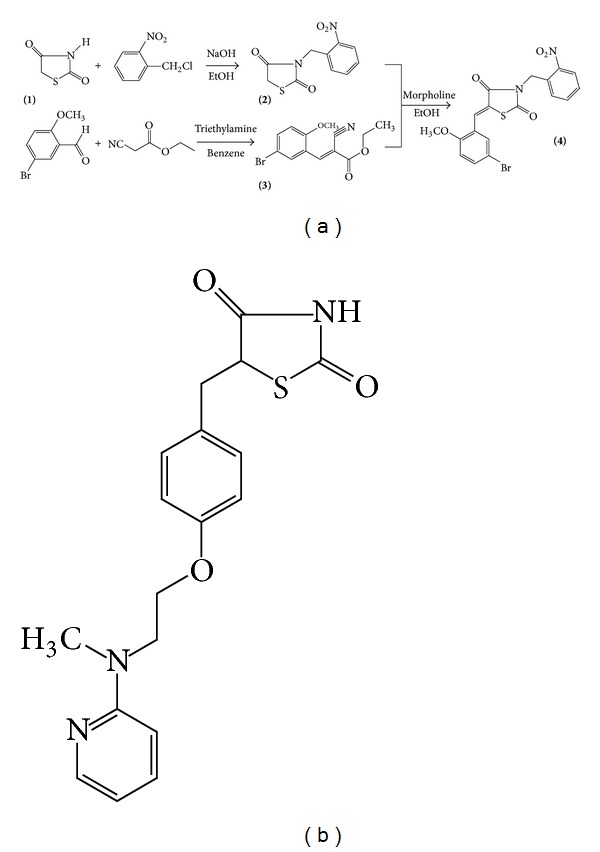
(a) Synthetic route for *Z*-5-(5-bromo-2-metoxy-benzylidene)-3-(2-nitro-benzyl)-thiazolidine-2,4-dione (TM17). (b) Rosiglitazone chemical structure.

The 5-(5-bromo-2-methoxy-benzylidene)-3-(2-nitro-benzyl)-thiazolidine-2,4-dione TM17 (**4**) was prepared by a nucleophilic Michael addition of the benzyl-thiazolidine-2,4-dione (**2**) and the substituted cyanoacrylate (**3**) to obtain the benzylidene-thiazolidine-2,4-dione TM17 (**4**) as described in Figure 1. The thiazolidine-2,4-dione (1) was N-(3)-alkylated with the 2-nitro-benzyl chloride in the presence of sodium hydroxide in hot ethanol leading to formation of the intermediate 3-(2-nitro-benzyl)-thiazolidine-2,4-dione (2). The 3-(5-bromo-2-methoxy-phenyl)-2-cyano-acrylic acid ethyl ester (**3**) was prepared by Knoevenagel condensation of ethyl cyanoacetic ester and 5-bromo-2-methoxy-benzaldehyde in the presence of triethylamine. The published chemical data on 3-(5-bromo-2-methoxy-phenyl)-2-cyano-acrylic acid ethyl ester [20] are not reported here. The benzylidene-thiazolidine-2,4-dione TM17 (**4**) was isolated in a single isomeric form, which was verified by TLC and NMR analysis. X-ray crystallographic studies and 13CNMR have demonstrated a preference for the *Z* configuration for 5-benzylidene-thiazolidinones [21–23]. The melting point was measured in a capillary tube on Quimis 340.23 apparatus. Thin layer chromatography was performed on silica gel plates (Merck 60F254). Infrared spectra of 1% KBr pellets were recorded on a Bruker IFS66 spectrometer. 1HNMR spectra were recorded on a Varian Unity Plus 300 MHz spectrophotometer in DMSO-d_6_ as the solvent, with tetramethylsilane as the internal standard. Mass spectra were recorded on an LCMS-IT-TOF Shimadzu Liquid Chromatograph mass spectrometer in positive polarity. (*5Z*)-5-(5-Bromo-2-methoxy-benzylidene)-3-(2-nitro-benzyl)-thiazolidine-2,4-dione TM17: an equimolar (0.83 mMol) mixture of 3-(2-nitro-benzyl)-thiazolidine-2,4-dione (**2**) and 3-(5-bromo-2-methoxy-phenyl)-2-cyano-acrylic acid ethyl ester (**3**) dissolved in ethanol (10 mL) with morpholine (250 *μ*L) was heated at 50°C for 3 h. After cooling, the precipitated product was purified by flash column chromatography on silica with *n*-hexane/ethyl acetate, 8 : 2 as the eluent. C_18_H_13_BrN_2_O_5_S. Yield 30,58%. Mp 164°C. TLC (benzene : ethyl acetate, 9,5 : 0,5) Rf 0,72. ^1^H NMR (300 MHz, DMSO-d_6_) 3.91 (3H, s, OCH_3_), 5.17 (2H, s, NCH_2_), 7.16 (1H, d, *J* = 9 Hz, PhBrOCH_3_), 7.42 (1H, dd, *J* = 7.8, 1,2 Hz, PhNO_2_), 7.58 (1H, d, *J* = 2.09 Hz, PhBrOCH_3_), 7.58–7.63 (1H, m, PhNO_2_), 7.67–7.71 (1H, d, 8,7 Hz, PhBrOCH_3_), 7.71–7.76 (1H, m, PhNO_2_), 7.98 (1H, s, HC=), 8.12 (1H, dd, *J* = 8.4, 1.2 Hz, PhNO_2_). IR: 1733 and 1678 (C=O), 1598 (HC=). MS, ESI^+^ *m/z* 448, [M + Na]^+^ 471, [M + Na + 2]^+^ 472.96.

### 3.2. Cytotoxicity

For all conditions tested, cells presented a viability greater than 80%. On the highest dose tested, (100 *μ*M) TM17 showed a viability of 97.99 (±17.65) in splenocytes and 86.52 (±9.23) for PBMCs. The compound was also tested at 10 *μ*M in PBMCs and 250 *μ*M in splenocytes. The first showed 94,81 (±16,39) of viability and the second 84,3 (±2,9). This result confirms that TM17 is a nontoxic compound, even in high doses. 

### 3.3. The Role of TM17 in BALB/c Splenocytes

**Figure 2:**
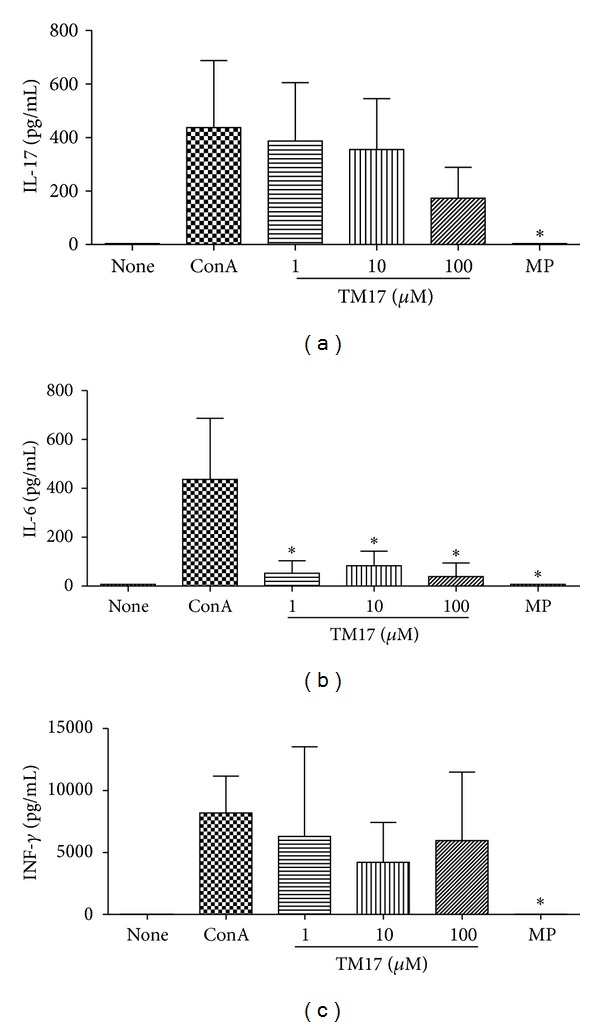
Evaluation of proinflammatory cytokines release inhibition by TM17 compound in splenocytes culture. (a) TM17 inhibits the release of IL17A in a dose-dependent manner. (b) IL6 was significantly inhibited by TM17 in all tested doses. (c) TM17 decreases IFN*γ* release mainly in 10 *μ*M. *P < 0.05.

In order to investigate whether TM17 treatment could modulate cytokines expression we first utilized BALB/c splenocytes. A dose-dependent reduction was observed in IL-17A level although the differences were not significant (Figure 2(a)). Regarding to IL-6 expression in all TM17 tested concentrations compared with ConA treated cells, there was a significant reduction in the production of this cytokine (*P* < 0.02) (Figure 2(b)). As for IL-17, there was a decrease in IFN-*γ* release in splenocytes treated with TM17 in a nonsignificative way (Figure 2(c)). IL-22 was also tested, but the levels were not detected in any tested condition. These results suggest that TM17 reduce IL-17A secretion that could be produced primarily by Th17 and *γδ*T cells [24] and INF-*γ*, secreted primarily by Th1 and NKT cells [25], thus acting on different immune cells. Another very interesting TM17 result is the reduction of IL-6 which participates in the Th17 phenotype polarization [26], suggesting that IL-6 secretion inhibition by TM17 could be associated with IL-17A reduced levels. 

### 3.4. The TM-17 Role in Peripheral Blood Mononuclear Cells from RA Patients and Healthily Donors

**Figure 3:**
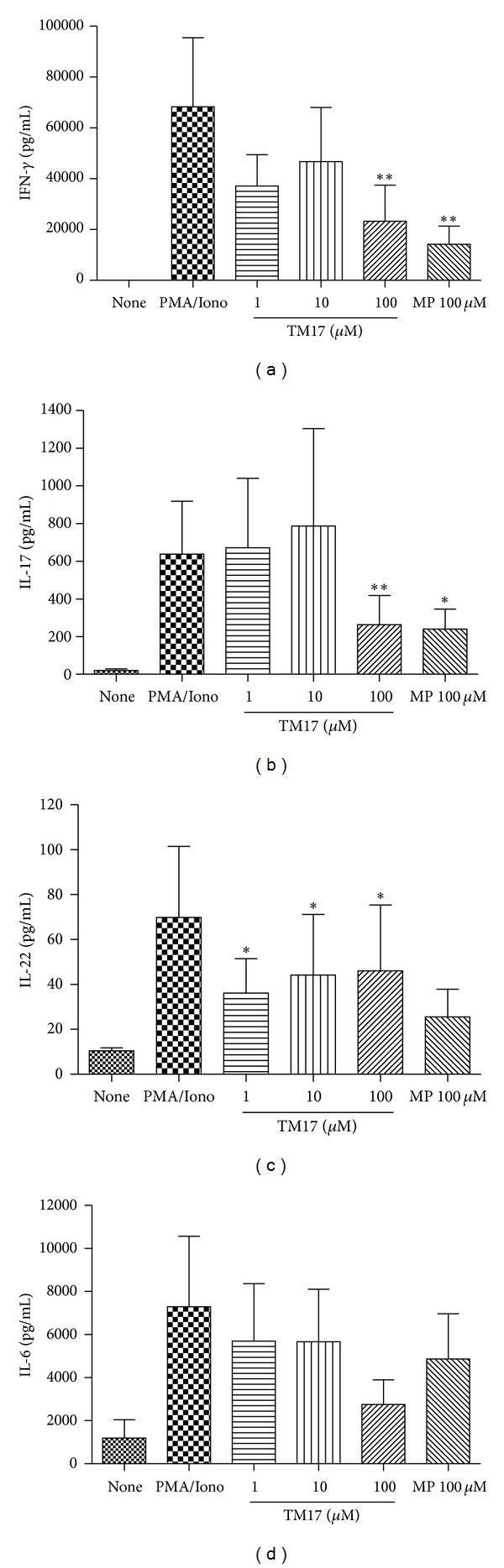
Evaluation of cytokines release inhibition by TM17 compound in PBMC culture from RA patients. (a) TM17 in 100 *μ*M as well as positive control (methylprednisolone) was able to decrease significantly INF-*γ* levels. The same pattern was observed for IL17A (b). (c) IL22 was significantly inhibited in all tested concentrations. (d) IL-6 was not significantly inhibited either by TM17 or by methylprednisolone. *P < 0.05,**P < 0.01.

**Figure 4:**
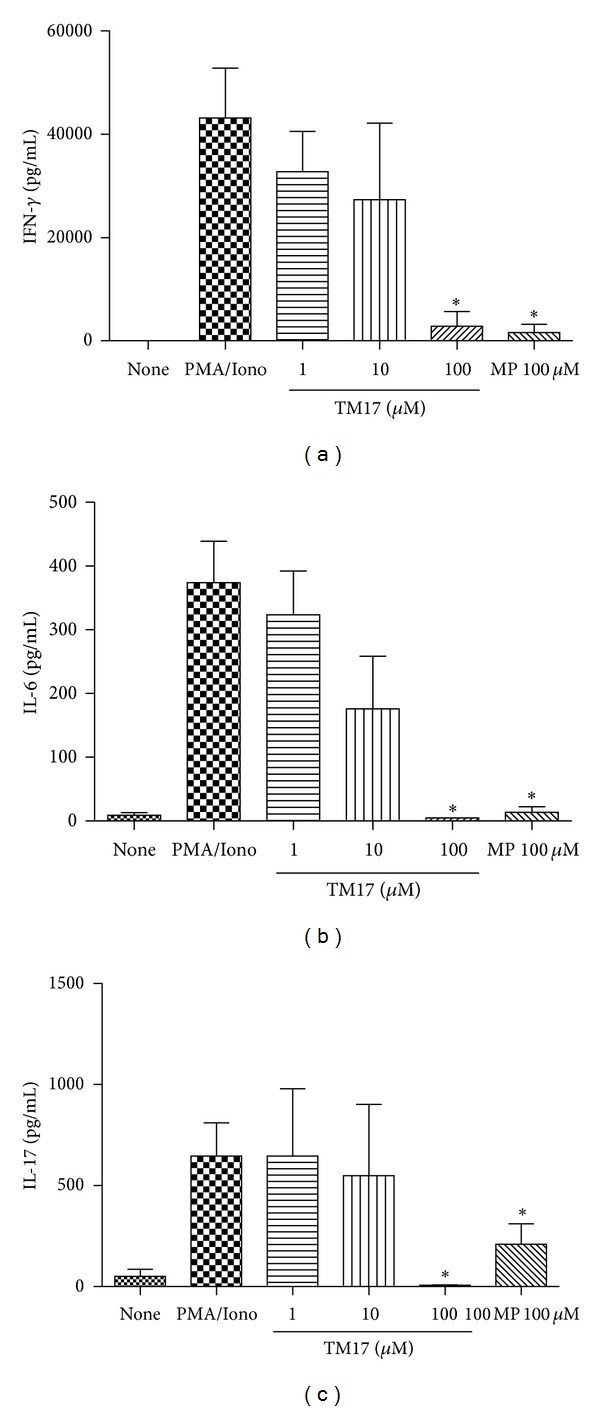
Evaluation of cytokines release inhibition by TM17 compound in PBMC culture from healthly donor. (a) TM17 in 100 *μ*M as well as positive control (methylprednisolone) was able to decrease significantly INF-*γ* levels. The same pattern was observed for IL-6 (b) and IL-17A (c). *P < 0.05.

To investigate the effect of TM17 treatment in IL-17A, IL-22, and IFN-*γ* releasing RA patients, supernatants were collected and assayed for these cytokines by ELISA. As shown in Figure 3, TM17 caused a reduction in a non-dose-dependent manner of IFN-*γ* (*P* = 0.0039) and IL-17A (*P* = 0.0078) but significant at 100 *μ*M when compared to PMA/IONO stimulated cells alone (Figures 3(a) and 3(b)). We also observed a significant IL-22 reduction after TM17 treatment at 100 *μ*M (*P* = 0.035), 10 *μ*M (*P* = 0.022), and 1 *μ*M (*P* = 0.022) differing from MP at 100 *μ*M (*P* = 0.058) when compared with PMA/IONO stimulated cells (Figure 3(c)). Although the findings were not statistically significant (*P* = 0.07), they show a tendency to decreased IL-6 levels secreted by PBMCs from patients with RA following TM17 at 100 *μ*M (Figure 3(d)). Tests with PBMCs from healthy donors were also conducted. However in these experiments the IL-22 levels were not detected in any condition analyzed. IFN-*γ* (Figure 4(a)) and IL-6 (Figure 4(b)) levels were twice lower, on average, than levels form RA patients PBMCs cultures; nevertheless the TM17 at 100 *μ*M retained its ability to inhibit both cytokines (*P* < 0.05). For IL17 (Figure 4(c)), the compound only retains the ability to significantly reduce the levels of this cytokine at 100 *μ*M (*P* = 0.031), suggesting that the compound preferentially inhibits the high levels of IL-17 secreted by RA patients cells. Ma and colleagues (2010) showed that besides the well-characterized anti-inflammatory activity of thiazolidinedione ring, the group methoxybenzylidene is also important in this activity [27]. Using a murine model of arthritis, they proved that the compound (*Z*)-5-(4-methoxybenzylidene) thiazolidine-2,4-dione inhibits the migration of macrophages and decreases the expression of proinflammatory cytokines such as TNF, IL1-*β*, and IL-6. In a recent study by our group [28] the compound 5-(4-benzylidene-methanesulfonyl)-3-(4-nitrobenzyl)-thiazolidine-2,4-dione also showed an antioxidant and anti-inflammatory activity, so these data together show that the methoxybenzylidene and nitrobenzyl groups could contribute with thiazolidine effect.

### 3.5. TM17 Modulate PPAR*γ* mRNA Expression

**Figure 5:**
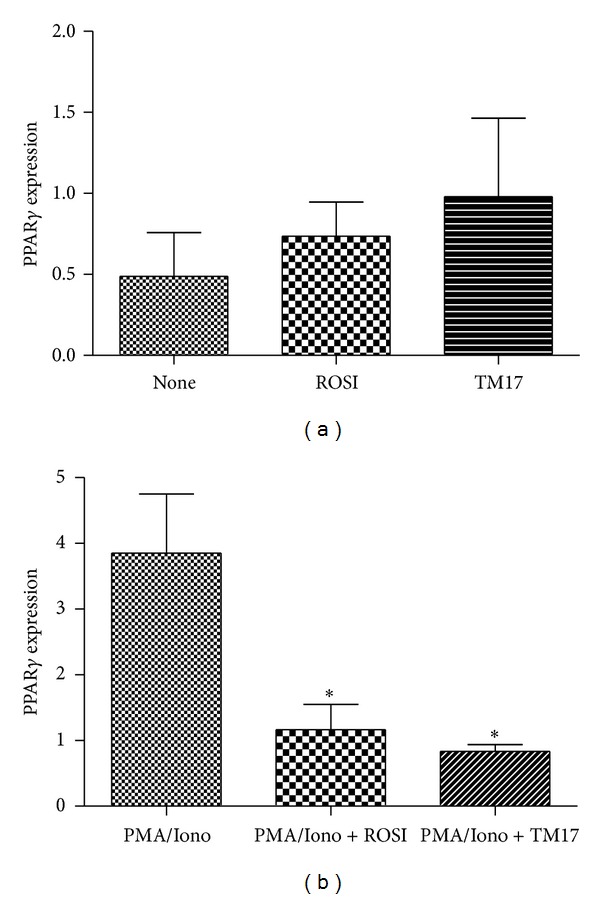
PPAR*γ* expression in PBMCs from healthy individuals. (a) PPARy mRNA fold increase in cells treated with rosiglitazone and TM17 compound at 100 *μ*M concentration. (b) Iono and PMA enhance PPAR*γ* expression, and TM17 and rosiglitazone reduce significantly PPAR*γ* mRNA in this condition. *P < 0.05.

RA is a systemic inflammatory disease of joints characterized by monocytes/macrophages infiltration, B and T cell activation, autoantibody formation, and production of several cytokines and matrix metalloproteinases (MMP)s, causing persistent inflammation [3]. Peroxisome proliferator-activated receptor-gamma (PPAR*γ*) plays a relevant anti-inflammatory role in various diseases, including AR [7]. Accordingly, in order to access PPAR*γ* modulation by TM17 since this compound is a TZD derivative, we evaluate PPAR*γ* mRNA expression in PBMCs from healthy individuals exposed six hours to TM17. The already known PPAR*γ* agonist rosiglitazone was used as positive control. As shown in Figure 5(a), both drugs at 100 *μ*M induce PPAR*γ* expression in PBMCs, but TM17 increases PPAR*γ* expression more expressively. Palma and coworkers (2012) also demonstrated that the PPAR*γ* agonists 15d-PGJ, methotrexate, and methylprednisolone increase expression of the receptor in cells isolated from healthy donors [8]. Furthermore, the same study showed that patients with rheumatoid arthritis have increased PPAR*γ* expression compared to healthy subjects and receptor expression may be associated with a better prognosis. Although the results suggest that TM17 act as PPAR*γ* modulator, further studies should be conducted to confirm possible role of this new compound as PPAR*γ* agonist and also other mechanisms of action independent of PPAR*γ*. 

We also analyzed *in vitro* the ability of PMA/IONO (used as standard stimulus) to affect directly PPAR*γ* expression, in the absence or presence of PPAR*γ* agonists. As shown in Figure 5(b), PMA/IONO, a strong inflammatory stimulus, increased PPAR*γ* expression (*P* = 0.035). On the opposite, when rosiglitazone or TM17 was added in the system, these compounds inhibit inflammation and consequently reduce PPAR*γ* mRNA levels. TM17 significantly reduced PPAR*γ* expression in PBMCs stimulated with PMA/IONO (*P* = 0.021). Interestingly, this decrease was higher compared to the positive control rosiglitazone (*P* = 0.04). Our results are in agreement with Klotz and coworkers findings [29]. Their studies study showed that in the experimental model of multiple sclerosis, PPAR*γ*-mediated T-cell-intrinsic molecular mechanism selectively controls Th17 differentiation by inhibition of TGF-beta/IL-6-induced expression of ROR*γ*t in T cells. The authors also concluded that PPAR*γ* represents a promising molecular target for specific immunointervention in Th17-mediated autoimmune diseases. 

## 4. Conclusion

This work shows that PBMCs from RA patients under TM17 treatment present a significant reduction in IL-17A, IL-22, and IFN-*γ* expression but not IL-6, unlike mice splenocytes where the compound significantly inhibited IL-6 but not IL-17A and IFN-*γ*. The compound also enhanced PPAR*γ* mRNA expression indicating this new compound as promisor in inflammatory and autoimmune diseases treatment, mainly by reducing IL-17A levels in RA cells. 

## Figures and Tables

**Table 1:** Demographic, clinical, and laboratory presentation of the patients with RA.

Number of patients	9
*Age (years) *	
Mean (range)	52.6 (30–69)
*Sex *	
Female/male	9/0
*Disease duration (years) *	
Mean (range)	6 years (0.1–16.2)
*Rheumatoid factor *	
Positive/negative	8/1
*Treatment *	
Nonsteroidal anti-inflammatory drugs	1
Steroids	8
Methotrexate	4
Leflunomide	2
Antimalarial agents	1
Biologic therapy	—
*Disease activity *	
DAS28	
Clinical remission	—
Mild disease	4
Moderate disease	2
Severe disease	3
CDAI	
Clinical remission	—
Mild disease	—
Moderate disease	5
Severe disease	4
